# Phenotyping the Responses to Systemic Corticosteroids in the Management of Asthma Attacks (PRISMA): protocol for an observational and translational pilot study

**DOI:** 10.1136/bmjresp-2023-001932

**Published:** 2023-11-08

**Authors:** Carlos Andrés Celis-Preciado, Simon Leclerc, Martine Duval, Dominic O. Cliche, Pierre Larivée, Samuel Lemaire-Paquette, Simon Lévesque, Andréanne Côté, Philippe Lachapelle, Simon Couillard

**Affiliations:** 1 Faculté de Médecine et des Sciences de la Santé, Université de Sherbrooke, Sherbrooke, Quebec, Canada; 2 Internal Medicine-Pulmonary Unit, Faculty of Medicine, Hospital Universitario San Ignacio, Pontificia Universidad Javeriana, Bogota, Colombia; 3 Laboratoire de Microbiologie, CIUSSS de l’Estrie–CHUS, Sherbrooke, Quebec, Canada; 4 Department of Medicine, Faculty of Medicine, Centre de Recherche de l’Institut universitaire de cardiologie et de pneumologie de Québec-Université Laval, Quebec City, Quebec, Canada

**Keywords:** Allergic lung disease, Asthma, Asthma Mechanisms, Cytokine Biology, Inflammation, Viral infection, Exhaled Airway Markers, Respiratory Function Test

## Abstract

**Introduction:**

Asthma and its associated exacerbation are heterogeneous. Although severe asthma attacks are systematically prescribed corticosteroids and often antibiotics, little is known about the variability of response to these therapies. Blood eosinophils and fractional exhaled nitric oxide (FeNO) are type 2 inflammation biomarkers that have established mechanistic, prognostic and theragnostic values in chronic asthma, but their utility in acute asthma is unclear. We speculate that the clinical and biological response to those treatments varies according to inflammometry and microbiological test results.

**Methods and analysis:**

An observational longitudinal pilot study with multimodal clinical and translational assessments will be performed on 50 physician-diagnosed ≥12-year-old asthmatics presenting with an asthma attack and 12 healthy controls, including blood eosinophil count (venous and point-of-care (POC) capillary blood), FeNO and testing for airway infection (sputum cultures and POC nasopharyngeal swabs). People with asthma will be assessed on day 0 and after a 7-day corticosteroid course, with home monitoring performed in between. The primary analysis will be the change in the forced expiratory volume in 1 s according to type 2 inflammatory status (blood eosinophils ≥0.15×10^9^/L and/or FeNO ≥25 ppb) after treatment. Key secondary analyses will compare changes in symptom scores and the proportion of patients achieving a minimal clinically important difference. Exploratory analyses will assess the relationship between clinical, lung function, inflammatory and microbiome parameters; satisfaction plus reliability indices of POC tests; and sex–gender variability in treatment response. Ultimately, this pilot study will serve to plan a larger trial comparing the clinical and biological response to systemic corticosteroids according to inflammatory biomarkers, offering valuable guidance for more personalised therapeutic strategies in asthma attacks.

**Ethics and dissemination:**

The protocol has been approved by the Research Ethics Committee of the CIUSSS de l'Estrie–CHUS, Sherbrooke, Quebec, Canada (#2023-4687). Results will be communicated in an international meeting and submitted to a peer-reviewed journal.

**Trial registration number:**

ClinicalTrials.gov Registry (NCT05870215).

**WHAT IS ALREADY KNOWN ON THIS TOPIC**

- Asthma exacerbations pose a substantial burden on healthcare systems and individuals. Despite existing knowledge, there are still gaps in our understanding of the precise mechanisms underlying asthma exacerbations, the role of biomarkers and the potential for more personalised treatment approaches.

**WHAT THIS STUDY ADDS**

- Phenotyping the Responses to Systemic Corticosteroids in the Management of Asthma Attacks (PRISMA) is an observational longitudinal pilot study with multimodal clinical and translational assessments that aims to stratify the clinical and biological responses to systemic corticosteroids in asthma attacks according to type 2 inflammatory biomarkers (blood eosinophils and fractional exhaled nitric oxide) and will explore the relationship between other clinical, inflammatory biomarkers/proteins, and microbiological parameters and treatment response.

**HOW THIS STUDY MIGHT AFFECT RESEARCH, PRACTICE OR POLICY**

- By conducting an extensive array of clinical and translational assessments, this study aims to provide a holistic view of asthma attacks, shedding light on their multifaceted nature and paving the way for a comprehensive, larger-scale clinical trial.

## Introduction

Asthma is a prevalent chronic respiratory disease affecting over 400 million people worldwide.^1^ Asthma attacks are loosely defined as deterioration of symptoms and/or lung function from baseline.^2^ In contrast, the more stringent classification of attack severity is based on treatment decisions: a severe episode requiring emergency room consultation and/or ≥3 days of oral corticosteroids (OCs) and/or hospitalisation.^3^ Severe asthma attacks cause morbidity, healthcare utilisation and avoidable deaths.^4^ Attacks are ‘red flags’^5^: a sentinel event and an opportunity for personalised asthma management.^6^ Despite growing evidence of heterogeneity of mechanisms driving asthma attacks, the standard of care in acute asthma has not changed for 30 years. It consists of a ‘one-size-fits-all’ treatment with OCs and often antibiotics.^7 8^

In stable severe asthma, the treatment paradigm is evolving towards targeting treatable traits^9^—a revolution launched after heterogeneity of stable severe asthma was established clinically^10^ and translationally.^11–13^ An important aspect of this paradigm is to guide therapeutic decisions based on clinical characteristics that predict greater treatment response. The most noteworthy application is the targeted use of anti-inflammatory medications in asthma characterised by a type 2 inflammatory phenotype, identified in the clinic using simple tests such as blood eosinophil count (BEC) and fractional exhaled nitric oxide (FeNO).^6 14^

BEC and FeNO are effective biomarkers for several reasons. First, they provide complementary mechanistic information on different immune compartments involved in the pathogenesis of asthma. Blood eosinophilia is predominantly caused by circulating interleukin (IL)-5 activity and reflects the systemic component of type 2 inflammation,^15 16^ whereas FeNO elevation reflects the airway epithelial component mediated by IL-13-mediated inducible nitric oxide synthase.^16–18^ Second, they have additive and independent value in predicting severe asthma attacks in control arm trial populations across mild,^19^ moderate^20^ and severe asthma.^21–23^ Third, BEC and FeNO predict treatment response: raised levels are associated with greater benefit from anti-inflammatory therapies in chronic asthma, be it low-dose inhaled corticosteroids in mild disease,^19^ higher-dose inhaled corticosteroids in moderate^20 24^ or type 2 targeted biologics in moderate-to-severe asthma.^25–27^ Importantly, these biomarkers are simple, non-invasive and accessible.^14^ Just as high blood pressure and cholesterol levels are regularly assessed to predict and prevent heart attacks, BEC and FeNO are emerging as airway equivalents to quantify the modifiable risk of asthma attacks.^23 27 28^

The critical remaining questions are (a) how airway signalling (reflected by FeNO) and an increased systemic eosinophil pool (reflected by BEC) relate to the underlying biological pathways that contribute to asthma exacerbation and (b) how the type 2 inflammatory phenotype impacts the response to acute anti-inflammatory corticosteroid treatment. An often-cited confounder is airway infection, which is difficult to diagnose when relying on conventional sputum cultures but can indicate either greater susceptibility to macrolide antibiotics^29 30^ or, conversely, can decrease the use of antibiotics (when a viral infection is diagnosed and/or no bacterial infection is identified).^31^

In acute asthma, several studies have documented the heterogeneity of attacks,^32–39^ with the presence of sputum inflammation characterised by elevated eosinophils^32 33 36–38^ or neutrophils.^34–38^ Importantly, these inflammatory phenotypes are indistinguishable from the point of view of initial symptoms or lung function.^38^ Other features are high BEC and elevated FeNO,^37^ increased levels of IL-8 in sputum^35 36^ or serum C reactive protein (CRP)^37^ or a different microbial flora related to FeNO values.^39^ However, treatment responses to acute systemic corticosteroid and antibiotic courses have not been related to the type 2 inflammatory phenotype and the presence of airway infection, respectively.

Concerning point-of-care (POC) measurements, it is noteworthy that capillary POC-BEC has been successfully studied in chronic airway diseases^40 41^ but not in asthma attacks; they are also approved by Health Canada and the US Food and Drug Administration (FDA).^40^ Furthermore, POC-FeNO technologies also exist, have been used for remote monitoring of FeNO, and are approved for use by Health Canada and the FDA.^41^

Finally, following the COVID-19 pandemic, we have increasing evidence supporting the relevance of early knowledge of viral and/or bacterial infection. Brendish *et al* previously showed that antibiotic use in acute airway disease dropped only once the physician had the results from the POC test, thus highlighting the importance of immediate notification of rapid multiplex testing.^31^ Improving the accessibility and affordability of POC inflammometry and microbiology testing in asthma is very likely to improve the situation for patients, physicians, manufacturers and pharmaceutical companies because it will optimise the management of the patients, identify those with type 2-driven worsening of the disease giving them better access to targeting medications and avoiding unnecessary antibiotics.^42^

The treatable trait success story of chronic asthma certainly makes a case for the reappraisal of our approach to acute asthma.^6 7 43^ Is it time for precision medicine to attack the problem of asthma attacks? A worthwhile question, considering that corticosteroid toxicities appear after the lifetime equivalent of only four bursts (≥1000 mg cumulative dose) of prednisone—that is, four severe asthma attacks^44^—and the increasing levels of resistance to antibiotics observed, mainly due to inadequate prescriptions.^7 29^ Biomarker-guided management could avoid harm to individual patients and society stemming from the inappropriate use of OCs and/or antibiotics due to overuse or without proper consideration of their potential risks and side effects.^44 45^ Simply stated, corticosteroid and antibiotic stewardship can and should be assisted by acute POC testing.

Based on previous knowledge from chronic stable asthma and reports that asthma attacks are heterogeneous events, we hypothesise that treatment responses to OCs in acute asthma vary according to the underlying inflammatory phenotype. Specifically, we speculate that events identified by raised BEC and/or FeNO benefit most from OCs and that events presenting with low type 2 biomarkers have a lesser objective clinical and biological anti-inflammatory response but perhaps higher rates of airway infection or dysfunction. If this proves to be the case, we will be in a solid position to move forward with a randomised clinical trial comparing biomarker-directed OC use with the current ‘one-size-fits-all’ approach. An important secondary hypothesis of our study is that POC inflammatory and microbiological assessments in acute asthma are possible, acceptable and reliable. The ramifications of such findings would be to plan a clinical trial assessing biomarker-guided asthma attack management in primary care.

## Methods and analysis

### Study design

**Figure 1:**
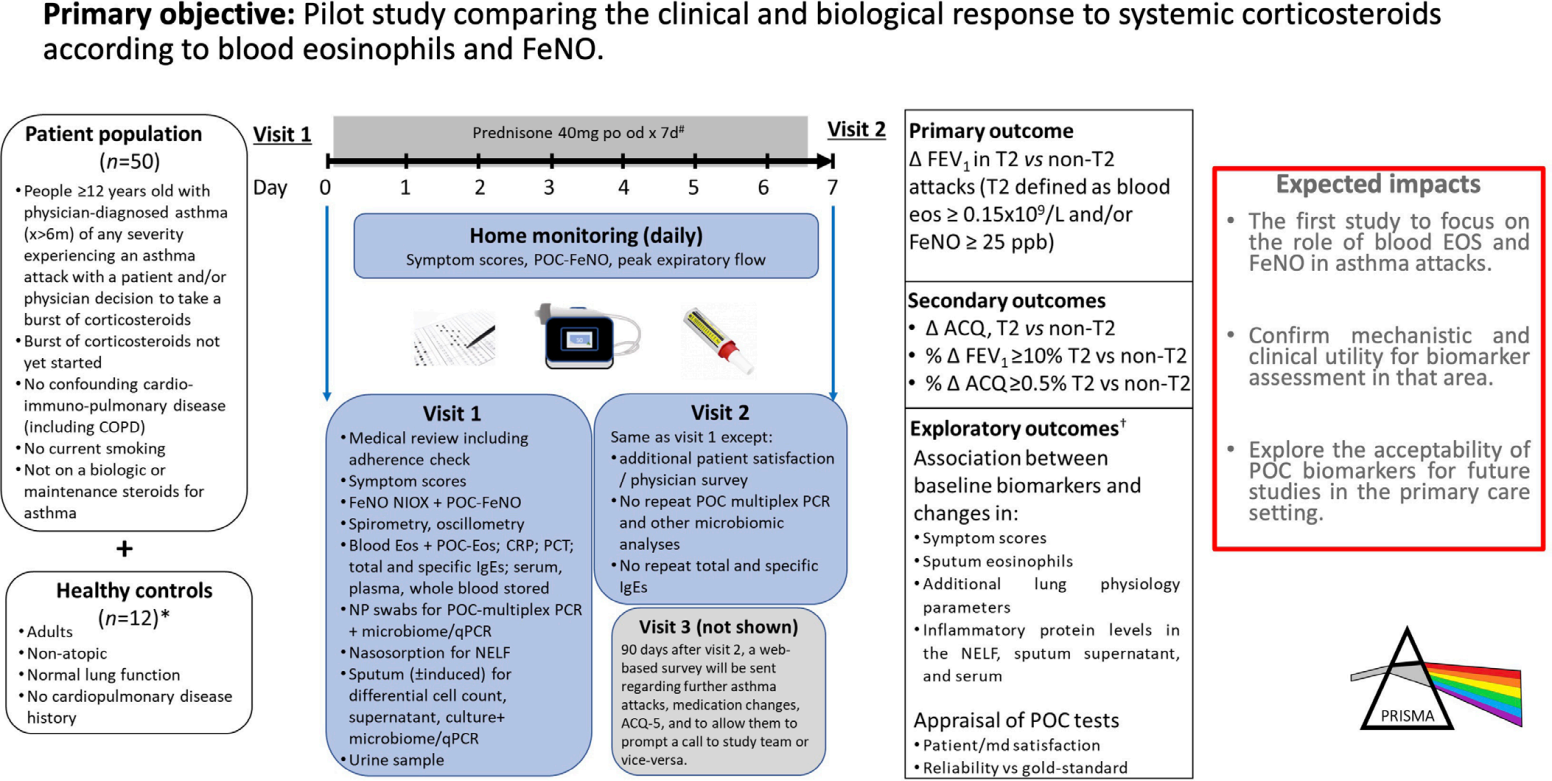
Study design, methods overview and expected impacts of the PRISMA asthma attack pilot study. Our hypothesis is that asthma attacks are heterogeneous events to which objective treatment responses following systemic corticosteroids vary according to the underlying inflammatory phenotype. *Healthy controls do only visit 1. #Concomitant antibiotic use will only be considered if: C reactive protein (CRP) >20 mg/L, procalcitonin (PCT) >0.25 µg/L or positive sputum to a significant bacterial pathogen. †Refers to the text for a full list of exploratory outcomes. ACQ-5, five-item Asthma Control Questionnaire; COPD, chronic obstructive pulmonary disease; Eos, eosinophil; FeNO, fractional exhaled nitric oxide; FEV_1_, forced expiratory volume in 1 s; NELF, nasal epithelial lining fluid; NP, nasopharyngeal; od, once daily; po, orally; POC, point-of-care; PRISMA, Phenotyping the Responses to Systemic Corticosteroids in the Management of Asthma Attacks; qPCR, quantitative PCR.

We planned an observational longitudinal pilot study with before/after multimodal assessments plus home monitoring (figure 1). The study will be mainly conducted at the Centre Hospitalier Universitaire de Sherbrooke’s research centre, with a secondary site to open at the Institut Universitaire de Pneumologie et Cardiologique de Québec (Québec, Canada) if needed for recruitment purposes. This is not a clinical trial; OCs are the standard treatment for asthma attacks and will be administered as required.

### Participants

People ≥12 years old with physician-diagnosed asthma since >6 months experiencing an asthma attack as defined by GINA (episode characterised by a progressive increase in symptoms of shortness of breath, cough, wheezing or chest tightness that represent a change from the patient’s usual status)^2^ with a patient and/or physician decision to initiate a burst of OCs (but not yet started) will be assessed within 24 hours on weekdays after a screening telephone call. Exclusion criteria are a SARS-CoV-2-positive event, asthma treated with a monoclonal antibody or maintenance OCs, current smoking, significant overlapping cardiopulmonary disease (including chronic obstructive pulmonary disease, defined as age >40 years old AND persistent airflow limitation with forced expiratory volume in 1 s (FEV_1_)/forced vital capacity <0.7 AND >10 pack-year smoking history or α-1-antitrypsin deficiency), a confounding immunological state, ongoing pregnancy or contraindication to corticosteroids.

Although we will assess asthmatics with a history of physician-diagnosed asthma, patients included in the primary analysis and counting toward the target sample size will need to have objective proof of asthma as defined by international guidelines^2^ either based on the medical record or with a clinically required follow-up test ordered by the study respirologist and performed after study participation. Unconfirmed asthmatics data will be reported but not used for the primary analysis.

Non-atopic, non-smoking healthy volunteers with normal spirometry and no history of lung disease will form the control cohort. All participants (±their parent or legal tutor) must provide informed consent for the study protocol. Patients who cannot or refuse to participate in the diary card data collection for the home monitoring period will still be able to participate in all other study procedures.

### Study visits

Visit 1 will be on day 0 of the asthma attack, with oral prednisone issued by the community pharmacy (40 mg×7 days) (figure 1). Concomitant antibiotic use will only be considered for patients with ≥1 of the following criteria: CRP >20 mg/L, procalcitonin >0.25 µg/L (when available), or positive sputum culture and/or multiplex POC test for a significant bacterial pathogen. Healthy controls will attend only visit 1. Multimodal assessments performed at study visits lasting ~2 hours are detailed in table 1. In visit 1, these comprise:

- Medical history and examination, including vital signs, inhaler technique and adherence check (using prescription refills of community pharmacy), five-item Asthma Control Questionnaire (ACQ-5),^46 47^ dyspnoea rated on the modified Medical Research Council Scale,^48^ Visual Analogue Scale for respiratory symptoms, Pittsburgh Vocal Cord Dysfunction Index,^49^ Nijmegen Questionnaire,^50^ Hospital Anxiety and Depression Scale,^51^ chest X-ray.
- FeNO measurement (NIOX VERO, Circassia) and pre/post-bronchodilator oscillometry (Tremoflo C-100, Thorasys Thoracic Medical Systems) and spirometry plus peak expiratory flow.
- Peripheral blood tests (complete blood count with differential, serum CRP, total and specific serum IgE, biobank), POC-BEC (Sight OLO, Sight Diagnostics), urine sample (creatinine, biobank), nasosorption with nasal epithelial lining fluid (NELF), nasal swab for the ultrarapid ID NOW (Abbott Industries) SARS-CoV-2 molecular test, nasopharyngeal swab for rapid multiplex PCR using the BIOFIRE Respiratory 2.1 (RP2.1) Panel (bioMérieux). If no spontaneous sputum is available, following negative COVID-19 testing and post-bronchodilator FEV_1_ > 60% predicted value or >1.5 L, induced sputum collection with hypersaline nebulisation would then be performed, as done in a previous asthma attack study.^37^

**Table 1:** Study visit overview

Visit	Screening	0	1–6	7	97
Location	Telephone	Clinic	Home	Clinic	Virtual±telephone
Consent	Given	Signature			
Inclusion/exclusion criteria overview	x	x			
SARS-CoV-2 test (ID NOW)		x		x	
Medical history		x		x	x
Physical examination by doctor		x			
Inhaler technique check		x			
Adherence check (call to community pharmacy)		x			
Check if patient picked up prednisone tablets			x		
Review of test results by doctor				x	±
Questionnaires					
Medical Research Council dyspnoea scale		x		x	
Asthma Control Questionnaire (5 items)		x	x	x	x
Visual Analogue Scale of asthma symptoms (6 items)		x		x	
Pittsburgh Vocal Cord Dysfunction Index		x		x	
Nijmegen Questionnaire		x		x	
Hospital Anxiety and Depression Scale		x		x	
Survey of % of home tests monitored				x	
Satisfaction questionnaire about point-of-care biomarker measurements: participant				x	
Satisfaction questionnaire about point-of-care biomarker measurements: letter and email sent to treating doctor				x	
Respiratory physiology					
FeNO measurement (NIOX)		x		X	
Teaching of FeNO measurement		x			
FeNO measurement		x	x	x	
Teaching of peak expiratory flow measurement		x			
DEP measurement		x	x	x	
Capillary blood eosinophils: sight OLO measurement		x		x	
Force spirometry—pre-bronchodilator		x		x	
Forced oscillation—pre-bronchodilator					
Salbutamol 400 µg		x		x	
Force spirometry—post-bronchodilator		x		x	
Forced oscillation—post-bronchodilator					
Inflammometry					
Hospital viral multiplex PCR (BIOFIRE on nasopharyngeal swab)		x		x	
Nasosorption		x		x	
Nasal cytology brushes		x		x	
Induced or spontaneous sputum collection for differential cell count+supernatant, plugs and cell pellet for biobank		x		x	
Induced or spontaneous sputum collection for hospital microbiology analysis		x		x	
Hospital blood tests (1 EDTA tube, 1 heparin tube): complete blood count with differential, C reactive protein		x		x	
Allergy testing (hospital blood tests; 1 tube) for total serum IgE, specific serum IgE: grass mix, weed mix, tree mix, mould mix, house dust mites *Dermatophagoides pteronyssinus* and *D. farinae*, *Aspergillus fumigatus*		x			
Research laboratory blood tests: 1 EDTA, 4 heparin, 2 serum, 1 whole blood for research laboratory (biobank)		x		x	
Chest X-ray		x			
Urine sample; urine creatinine for hospital; sample frozen for research laboratory (biobank)		x		x	

FeNO, fractional exhaled nitric oxide.

Visit 2 will additionally include a survey of adverse events relating to corticosteroids, patient satisfaction questionnaires and permission to contact the patient’s primary care provider for a physician survey about POC testing.

Visit 3 is a web-based survey reviewing the number of subsequent exacerbations, medication changes and ACQ-5 score and allowing the patient to prompt a call from the study team (or vice-versa if the study team has a test to follow up on).

All study visits will occur in the morning to avoid the confounding effect of circadian variation of type 2 inflammatory biomarkers.^51–53^

### Home monitoring

Between visits 1 and 2, patients will be invited to complete daily symptom scores, FeNO and peak expiratory flow measurements via a paper diary card.

### Follow-up visit

Ninety days after visit 2, patients will be contacted by email to complete an internet form containing ACQ-5, peak flow measurement and questions about asthma management.

### Data and sample management

Individual participant data will be collected prospectively and managed using REDCap electronic data capture tools hosted at the Université de Sherbrooke.^54 55^ Frozen samples will be anonymised and logged prospectively in a freezer map.

### Specimen processing and analysis

**Figure 2:**
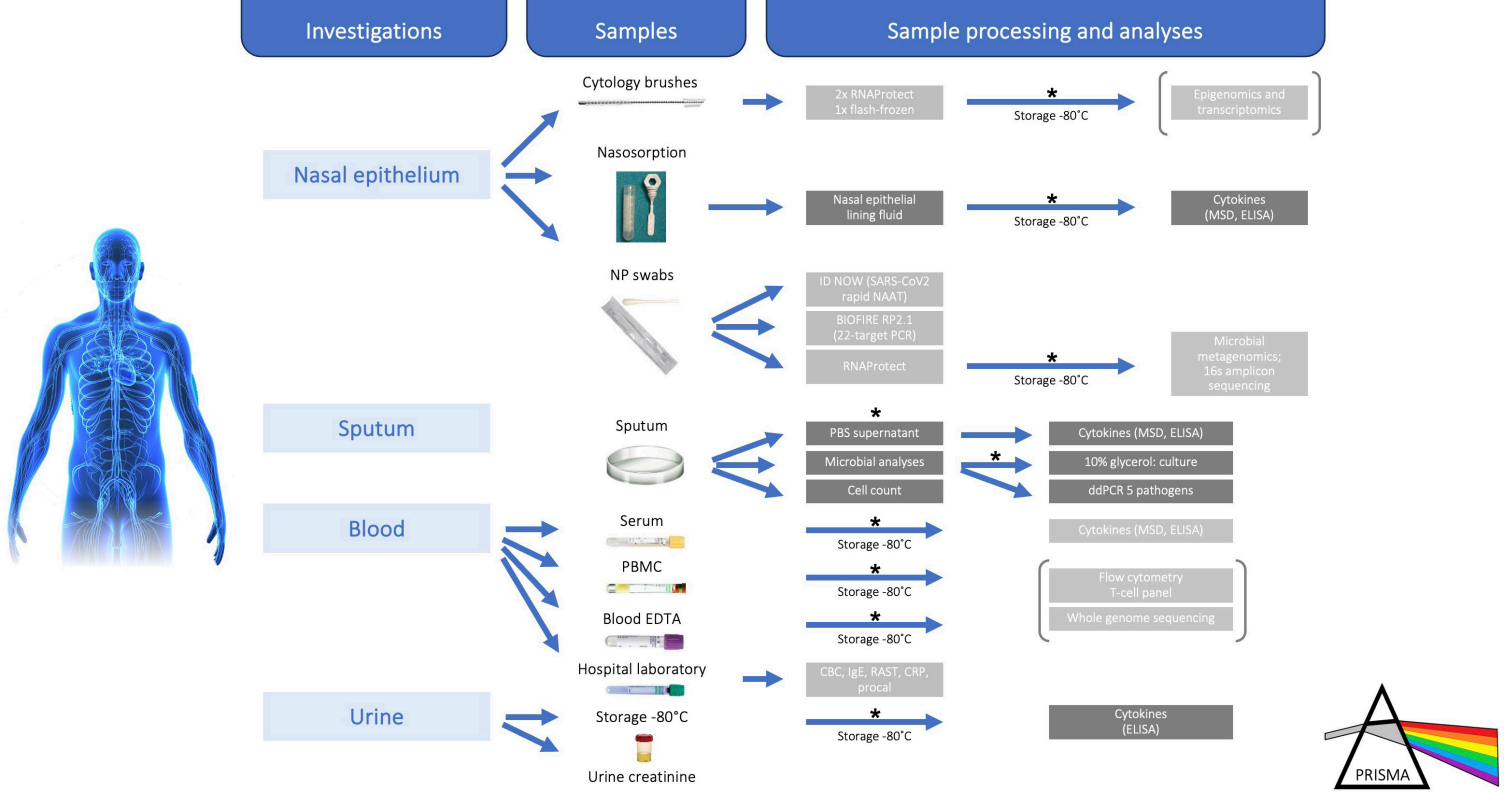
Multimodal and multicompartment sampling (biobanking) of highly phenotyped patients experiencing an asthma attack. In addition to many samples being analysed within the hospital laboratory, samples will be *biobanked within the Quebec Respiratory Health Research Network (QRHRN) biobank infrastructure (www.biobanque.ca). The collaborative nature of the biobank will allow further analyses and collaboration among QRHRN-affiliated academic research centres. Brackets indicate analyses not currently funded. CBC, complete blood count; CRP, C reactive protein; ddPCR, droplet digital PCR; MSD, Meso Scale Discovery; NAAT, nucleic acid amplification test; NP, nasopharyngeal; PBMC, peripheral blood mononuclear cell; PBS, phosphate-buffered saline; PRISMA, Phenotyping the Responses to Systemic Corticosteroids in the Management of Asthma Attacks; RAST, radioallergosorbent test; RP2.1, Respiratory 2.1.

Due to multimodal biobanking (figure 2), the study is closely associated with—but not dependent on—the local Quebec Respiratory Health Research Network biobank (www.biobanque.ca). Nasosorption strips will be spun with NELF eluted from a plastic mesh and then frozen (−80°C).^56^ Sputum will be mixed with Dulbecco’s phosphate-buffered saline (DPBS) and supernatant frozen before differential cell counting, as previously described.^57^ Whole blood will be processed into serum, buffy coat, primary blood mononuclear cell isolates, and whole blood samples and preserved frozen. Nasal swabs will be analysed immediately using the ultrarapid ID NOW molecular test to rule out SARS-CoV-2. One nasopharyngeal swab on visit 1 will be analysed using a POC multiplex PCR for 22 respiratory viruses and bacteria (BIOFIRE RP2.1 Panel) to identify infectious contributors to the attack, while the other nasopharyngeal brushes plus sputum plugs will be RNA stabilised then frozen (2× in RNA-Protect and 1× flash frozen). If sufficient sputum is produced at visit 1, a sputum plug will be sent for conventional bacterial culture, and up to three other plugs will be frozen (1× with dithiothreitol-DTT, 1× dry, 1× in 10% glycerol). Urine samples will be frozen.

At the end of the study, patient NELF, sputum DPBS-eluted supernatant, serum and urine will be thawed for inflammatory protein level measurements performed by multiplex electrochemiluminescent assays (Meso Scale Discovery, Meso Scale Diagnostics: Eotaxin-3, interferon-γ, IL-4/5/13/33, TARC, TSLP, tumour necrosis factor) and ELISA (sputum supernatant and urine cysteinyl leukotrienes corrected for creatinine). DNA and RNA will be extracted from nasopharyngeal brushes and sputum plugs. Quantitative digital droplet PCR, 16S ribosomal amplicon sequencing and shotgun metagenomics analysis will be used to detect and identify bacteria present in the samples. The 16S amplicon and shotgun metagenomics libraries will be sequenced using an Illumina Nextseq500 sequencer (Illumina).

### Outcome measures

#### Primary outcome

1. Change in the FEV_1_ between visits, with particular emphasis on a comparison based on the presence or absence of type 2 inflammatory events within the patient cohort. Specifically, we will classify type 2 high events on the basis of visit 1 BEC ≥0.15×10^9^ cells/L and/or FeNO ≥25 ppb.

#### Secondary outcomes

1. Change in ACQ-5 between visits according to type 2 inflammatory status.
2. The proportion of patients who achieve a minimal clinically important difference (MCID) ≥10% for FEV_1_ and ≥0.5 points for ACQ-5, according to the presence of type 2 high or type 2 low events.^58^ The magnitude of response will be rated as poor, moderate or good if the change is <1×, ≥1× or ≥2× the MCID values, respectively.

#### Exploratory outcomes

1. The continuous association between baseline biomarkers (BEC and FeNO) and changes in symptom scores, sputum inflammometry, additional lung physiology parameters (impulse oscillometry), inflammatory mediators (type 2 cytokines, chemokines, alarmins, eosinophils) and microbiome diversity across different immune compartments (nasal epithelium, induced sputum, serum, urine).
2. Comparative agreement and reliability of gold-standard BEC (hospital laboratory) versus POC test (Sight OLO).
3. An appraisal of POC biomarkers using patient satisfaction scores and primary care physician surveys.
4. A sensitivity analysis on all of the above assessing how sex at birth, gender (self-identified: female, male, other specify) and race (self-identified)^59^ influence treatment responses.

### Statistical analyses

Descriptive statistics will be presented for the complete sample and separated by groups (ie, frequencies and percentages for categorical, mean, and SD or median and IQR based on the distribution). The normality of variables will be assessed visually using Q–Q plots and histograms. Demographics will be compared between asthmatics and healthy controls using unpaired Student’s t-tests (Mann-Whitney) for continuous variables and Χ^2^ (Fisher’s exact test) for categorical variables. Due to the small sample size, no imputation methods are planned a priori for missing data. Frequencies and percentages of missing will be presented, and listwise deletion will be used for concerned analyses.

We will employ an unpaired two-sample t-test (Mann-Whitney U test if non-parametric) to assess the change in FEV_1_ (primary outcome) and ACQ-5 (key secondary outcome) between visits 1 and 2 according to type 2 inflammatory status. Additionally, to assess whether the observed change in FEV_1_ within subgroups defined by type 2 high events is clinically meaningful, we will employ a Χ^2^ test to compare the proportion of patients achieving the MCID between the two groups, using an MCID of 10% relative change in FEV_1_ and 0.5 points in ACQ-5. Finally, the above results will allow for a power calculation to plan a randomised controlled trial on key outcomes.

Exploratory analyses will include an association of the outcome measures with type 2 inflammatory mediators at visit 1 using further multivariable models. To consider the influence of potentially confounding variables, simple and multiple linear regression models will be performed for both independent variables (BEC and FeNO). Covariates considered are age, sex at birth, body mass index, atopic status, serum IgE, Asian race, nasal polyposis,^21^ smoking history, Charlson index, treatment intensity at baseline, exacerbation history in the past 12 months and ACQ-5. Only confounders associated (p<0.1) with FEV_1_ will be included in the final multivariable model. A linear mixed-effects analysis of the longitudinal improvements in parameters measured at home (FeNO, peak expiratory flows and symptom scores) according to baseline FeNO and BEC.

Patient satisfaction scores, physician surveys and the percentage of tests completed during the home monitoring period will be analysed descriptively. An intraclass correlation coefficient (two-way mixed model for absolute agreement, single measures) plus Bland-Altmann fixed bias estimate for gold standard (venous BEC performed in the laboratory) versus POC (capillary BEC via SightDx OLO) will be computed with 95% CIs.

In both primary and secondary outcomes, we will explore how treatment response to OCs may be influenced by sex, gender and diversity by conducting univariable and multivariable sensitivity analyses on those factors while also controlling for the type 2 inflammatory status. We will also perform a sensitivity analysis, disaggregating all statistical outputs by sex. Results will be presented using interaction plots.

All statistics will be analysed with a two-sided α of 0.05 and, when multiple inflammatory mediators are tested, by controlling for a false discovery rate <0.05.^60^ Statistical analyses will be performed with R V.4.1.2 (R Foundation) and GraphPad Prism (GraphPad Software, USA).

### Sample size

For our primary analysis, assuming a two-sided α error of 0.05, a T2:non-T2 event ratio of 1:1 and a mean±SD population difference in δ FEV_1_ of 10±10%, n=46 will provide 90% of power to detect the difference in our primary outcome on an unpaired two-sample t-test. According to a recent Canadian study that assessed the proportion of randomly selected adults with physician-diagnosed asthma who fulfil objective criteria to confirm the diagnosis of asthma, 18.3% of participants reporting a severe asthma attack in the past year (11 of 60) did not have asthma confirmed.^61^ We thus conservatively estimate that 20% of patients recruited will not count towards the n=50 needed for the primary analysis.

Including a cohort of 12 healthy control participants, each assessed during a single visit, is intended to enhance our ability to relativise demographics, lung function and inflammatory measurements observed in the study. Recruitment will continue until the target number of confirmed asthmatics and healthy controls has completed the study.

We acknowledge that the secondary and exploratory analyses will likely be underpowered and view these as exploratory but feasible within the proposed timeline of a pilot study serving to plan further clinical trials. Any significant finding for secondary and exploratory outcomes will require further confirmatory studies to support them.^62^

## Discussion

The proposed observational and translational before/after pilot study of asthma attacks across the range of asthma severities will be the first to focus on the role of BEC and FeNO and explore the clinical and biological factors affecting OC treatment response in acute asthma. We view our pilot study of POC measurements for assessing type 2 inflammation and airway infection as a stepping stone to plan a clinical trial allowing for biomarker implementation in the primary care setting. Biomarker-guided management could avoid harm to individual patients and society stemming from inappropriate use of OCs and/or antibiotics.

We expect events identified by raised BEC and/or FeNO to benefit most from corticosteroids, and POC tests to be useful theragnostic and mechanistic tools. Specifically, we expect increased BEC and FeNO to predict greater improvements in (a) FEV_1_ (primary outcome), (b) ACQ-5 scores (key secondary outcome), and (c) inflammatory mediators, symptoms, and other physiological aspects (exploratory outcomes). If the study hypothesis is confirmed—a phenotypical variability in treatment responses associated with type 2 airway inflammation—then the utility of BEC and FeNO could be studied to manage asthma attacks in a randomised controlled trial.

Three distinct studies in asthma attacks are underway, each with its unique objectives. ExCluSie-F (Phenotyping and Classifying Asthma Exacerbations; NCT05304039) is a prospective cohort that will explore the association between phenotypical characteristics and a composite outcome of treatment effectiveness. On the other hand, ‘APEX’ (Asthma: Phenotyping Exacerbations; NCT04293588) is a longitudinal cohort with participants who have experienced at least one asthma exacerbation in the previous year that aims to assess the proportion of patients displaying eosinophilic and non-eosinophilic phenotypes. Lastly, ‘BOOST’ (Recovery of Breakthrough Asthma Attacks Treated with Oral Steroids While on Monoclonal Antibody Therapy) is a prospective observational study involving adults on long-term biological treatment for asthma that will compare clinical recovery between individuals with high FeNO levels during their asthma attacks and those with low FeNO.^63^

PRISMA (Phenotyping the Responses to Systemic Corticosteroids in the Management of Asthma Attacks) Study offers a distinctive and comprehensive perspective in asthma exacerbation research compared with the studies above, as it stands out by exploring the vital and often less-understood components of asthma exacerbation: their response to standard medications, the utility of BEC and FeNO in acute settings, and the potential role of microbial infections in exacerbation dynamics. PRISMA can also be viewed as instrumental in participating and/or launching further studies about asthma attacks—a new and exciting frontier for biological therapies (eg, BeNReX (NCT04102800134), ABRA (NCT04098718135)).

If the POC test results prove reliable and are also acceptable to patients and their primary care providers, the table will be set for a placebo-controlled trial, such as being done in chronic obstructive pulmonary disease.^64–66^

The main potential pitfall is the unknown proportion of type 2 high versus type 2 low events in our sample, which ranges from mild-to-severe asthma. In severe asthma, approximately 80% of exacerbations are type 2 high,^38^ whereas in mild-to-moderate asthma, the proportion is unknown yet expected to be lower.^19 20^ However, we have planned our primary analysis on continuous variables and thus estimate that we will be able to pick up a signal of biomarker-based variability in acute corticosteroid responsiveness. The second major pitfall is our reliance on FEV_1_ as the primary outcome variable: type 2 inflammation has not always been linked with different FEV_1_ values.^12 38^ Third, we anticipate many patients may reach out to us either after starting OCs or due to a lack of response to a recent course. To ensure the exclusion of volunteers who have recently taken OCs, we have established a requirement that participants should not have taken a dose within the last 48 hours. Although this exclusion criterion may lead to a bias towards recruiting individuals with less severe events, as those requiring immediate OCs will not be included, we expect that most participants will be recruited through our specialised asthma clinics, which will facilitate the inclusion of severe asthmatics and individuals with more severe airway events. A fourth important limitation is that this is an observational study with a relatively small target sample size; the former does not preclude interesting mechanistic signals from being observed, yet we acknowledge the latter.

We are mindful of the safety concerns associated with conducting sputum induction after the pandemic. As a mitigation strategy, we will use nasosorption as a surrogate measure for assessing airway compartment inflammation. Additionally, we plan to implement rapid COVID-19 molecular testing at the beginning of each study visit to ensure the safety of our staff and increase the likelihood of conducting sputum induction.

To conclude, we have planned a study which is, to the best of our knowledge, the first to assess the variability in response to acute systemic therapies (corticosteroids and/or antibiotics) in asthma according to the inflammatory and microbial phenotypes. We hope that by providing this nuanced understanding, we can contribute to guiding the design of future biomarker-stratified randomised controlled trials that will be more finely tuned to the needs of individual patients with asthma.

## Data Availability

Data sharing not applicable as no datasets generated and/or analysed for this study. Not applicable.

## References

[1] To T, Stanojevic S, Moores G, et al. Global asthma prevalence in adults: findings from the cross-sectional world health survey. BMC Public Health 2012;12:204. 10.1186/1471-2458-12-204 22429515 PMC3353191

[2] Global Initiative for Asthma (GINA). Global strategy for asthma management and prevention (2022 updated). 2022. Available: https://ginasthma.org/

[3] Reddel HK, Taylor DR, Bateman ED, et al. An official American thoracic society/European respiratory society statement: asthma control and exacerbations: standardizing endpoints for clinical asthma trials and clinical practice. Am J Respir Crit Care Med 2009;180:59–99. 10.1164/rccm.200801-060ST 19535666

[4] Carroll W, Clayton S, Frost S, et al. “If it's 'only' asthma, why are children still dying?” Arch Dis Child 2020;105:494–8. 10.1136/archdischild-2019-318215 31871041

[5] Bush A, Pavord ID. Forthcoming UK asthma guidelines: an opportunity to improve asthma outcomes. Lancet 2021;398:1856–8. 10.1016/S0140-6736(21)02244-3 34656285

[6] Pavord ID, Beasley R, Agusti A, et al. After asthma: redefining airways diseases. Lancet 2018;391:350–400. 10.1016/S0140-6736(17)30879-6 28911920

[7] Ramakrishnan S, Couillard S. Antibiotics for asthma attacks: masking uncertainty. Eur Respir J 2021;58:2100183. 10.1183/13993003.00183-2021 34215662

[8] CONTROLLED trial of effects of cortisone acetate in status Asthmaticus; report to the medical research council by the subcommittee on clinical trials in asthma. Lancet 1956;271:803–6. 10.1016/S0140-6736(56)92241-3 13368522

[9] Agusti A, Bel E, Thomas M, et al. Treatable traits: toward precision medicine of chronic airway diseases. Eur Respir J 2016;47:410–9. 10.1183/13993003.01359-2015 26828055

[10] Haldar P, Pavord ID, Shaw DE, et al. Cluster analysis and clinical asthma phenotypes. Am J Respir Crit Care Med 2008;178:218–24. 10.1164/rccm.200711-1754OC 18480428 PMC3992366

[11] Woodruff PG, Modrek B, Choy DF, et al. T-helper type 2-driven inflammation defines major subphenotypes of asthma. Am J Respir Crit Care Med 2009;180:388–95. 10.1164/rccm.200903-0392OC 19483109 PMC2742757

[12] Pavord ID, Brightling CE, Woltmann G, et al. Non-eosinophilic corticosteroid unresponsive asthma. Lancet 1999;353:2213–4. 10.1016/S0140-6736(99)01813-9 10392993

[13] Wenzel SE, Schwartz LB, Langmack EL, et al. Evidence that severe asthma can be divided pathologically into two inflammatory subtypes with distinct physiologic and clinical characteristics. Am J Respir Crit Care Med 1999;160:1001–8. 10.1164/ajrccm.160.3.9812110 10471631

[14] Couillard S, Jackson DJ, Wechsler ME, et al. Workup of severe asthma. Chest 2021;160:2019–29. 10.1016/j.chest.2021.07.008 34265308

[15] Lambrecht BN, Hammad H, Fahy JV. The cytokines of asthma. Immunity 2019;50:975–91. 10.1016/j.immuni.2019.03.018 30995510

[16] Couillard S, Shrimanker R, Chaudhuri R, et al. Fractional exhaled nitric oxide nonsuppression identifies corticosteroid-resistant type 2 signaling in severe asthma. Am J Respir Crit Care Med 2021;204:731–4. 10.1164/rccm.202104-1040LE 34129808 PMC8521703

[17] Suresh V, Mih JD, George SC. Measurement of IL-13-induced iNOS-derived gas phase nitric oxide in human bronchial epithelial cells. Am J Respir Cell Mol Biol 2007;37:97–104. 10.1165/rcmb.2006-0419OC 17347445 PMC1899349

[18] Chibana K, Trudeau JB, Mustovich AT, et al. IL-13 induced increases in nitrite levels are primarily driven by increases in inducible nitric oxide synthase as compared with effects on arginases in human primary bronchial epithelial cells. Clin Exp Allergy 2008;38:936–46. 10.1111/j.1365-2222.2008.02969.x 18384429 PMC11934259

[19] Pavord ID, Holliday M, Reddel HK, et al. Predictive value of blood eosinophils and exhaled nitric oxide in adults with mild asthma: a prespecified subgroup analysis of an open-label, parallel-group, randomised controlled trial. Lancet Respir Med 2020;8:671–80. 10.1016/S2213-2600(20)30053-9 32171064

[20] Lee LA, Bailes Z, Barnes N, et al. Efficacy and safety of once-daily single-Inhaler triple therapy (FF/UMEC/VI) versus FF/VI in patients with inadequately controlled asthma (CAPTAIN): a double-blind, randomised, phase 3A trial. Lancet Respir Med 2021;9:69–84. 10.1016/S2213-2600(20)30389-1 32918892

[21] Kraft M, Brusselle G, FitzGerald JM, et al. Patient characteristics, biomarkers and exacerbation risk in severe, uncontrolled asthma. Eur Respir J 2021;58:2100413. 10.1183/13993003.00413-2021 34112734

[22] Shrimanker R, Keene O, Hynes G, et al. Prognostic and predictive value of blood eosinophil count, fractional exhaled nitric oxide, and their combination in severe asthma: a post Hoc analysis. Am J Respir Crit Care Med 2019;200:1308–12. 10.1164/rccm.201903-0599LE 31298922

[23] Couillard S, Laugerud A, Jabeen M, et al. Derivation of a prototype asthma attack risk scale centred on blood eosinophils and exhaled nitric oxide. Thorax 2022;77:199–202. 10.1136/thoraxjnl-2021-217325 34362839 PMC8762000

[24] Couillard S, Pavord ID. Fluticasone furoate: CAPTAIN of fluticasones in type 2 inflammatory asthma. Respirology 2022;27:184–6. 10.1111/resp.14213 35104914

[25] Castro M, Corren J, Pavord ID, et al. Dupilumab efficacy and safety in moderate-to-severe uncontrolled asthma. N Engl J Med 2018;378:2486–96. 10.1056/NEJMoa1804092 29782217

[26] Pavord ID, Korn S, Howarth P, et al. Mepolizumab for severe eosinophilic asthma (DREAM): a multicentre, double-blind, placebo-controlled trial. Lancet 2012;380:651–9. 10.1016/S0140-6736(12)60988-X 22901886

[27] Couillard S, Do WIH, Beasley R, et al. Predicting the benefits of type-2 targeted anti-inflammatory treatment with the prototype Oxford asthma attack risk scale (ORACLE). ERJ Open Res 2022;8:00570-2021. 10.1183/23120541.00570-2021 35141315 PMC8819242

[28] Couillard S, Steyerberg E, Beasley R, et al. Blood eosinophils, fractional exhaled nitric oxide and the risk of asthma attacks in randomised controlled trials: protocol for a systemic review and control arm patient-level meta-analysis for clinical prediction modelling. BMJ Open 2022;12:e058215. 10.1136/bmjopen-2021-058215 PMC8977743 35365539

[29] Taylor SL, Leong LEX, Mobegi FM, et al. Long-term azithromycin reduces haemophilus influenzae and increases antibiotic resistance in severe asthma. Am J Respir Crit Care Med 2019;200:309–17. 10.1164/rccm.201809-1739OC 30875247

[30] Taylor SL, Ivey KL, Gibson PG, et al. Airway abundance of haemophilus influenzae predicts response to azithromycin in adults with persistent uncontrolled asthma. Eur Respir J 2020;56:2000194. 10.1183/13993003.00194-2020 32366495

[31] Brendish NJ, Malachira AK, Armstrong L, et al. Routine molecular point-of-care testing for respiratory viruses in adults presenting to hospital with acute respiratory illness (respoc): a pragmatic, open-label, randomised controlled trial. Lancet Respir Med 2017;5:401–11. 10.1016/S2213-2600(17)30120-0 28392237 PMC7164815

[32] Baigelman W, Chodosh S, Pizzuto D, et al. Sputum and blood eosinophils during corticosteroid treatment of acute exacerbations of asthma. Am J Med 1983;75:929–36. 10.1016/0002-9343(83)90871-9 6650547

[33] Alfaro C, Sharma OP, Navarro L, et al. Inverse correlation of expiratory lung flows and sputum eosinophils in status asthmaticus. Ann Allergy 1989;63:251–4. 2774309

[34] Fahy JV, Kim KW, Liu J, et al. Prominent neutrophilic inflammation in sputum from subjects with asthma exacerbation. J Allergy Clin Immunol 1995;95:843–52. 10.1016/s0091-6749(95)70128-1 7722165

[35] Ordoñez CL, Shaughnessy TE, Matthay MA, et al. Increased neutrophil numbers and IL-8 levels in airway secretions in acute severe asthma: clinical and biologic significance. Am J Respir Crit Care Med 2000;161:1185–90. 10.1164/ajrccm.161.4.9812061 10764310

[36] Ghebre MA, Pang PH, Diver S, et al. Biological exacerbation clusters demonstrate asthma and chronic obstructive pulmonary disease overlap with distinct mediator and microbiome profiles. J Allergy Clin Immunol 2018;141:2027–36. 10.1016/j.jaci.2018.04.013 29709671 PMC5986707

[37] McDowell PJ, Diver S, Yang F, et al. The inflammatory profile of exacerbations in patients with severe refractory eosinophilic asthma receiving mepolizumab (the MEX study): a prospective observational study. Lancet Respir Med 2021;9:1174–84. 10.1016/S2213-2600(21)00004-7 33971168

[38] McDowell PJ, Busby J, Hanratty CE, et al. Exacerbation profile and risk factors in a type-2-low enriched severe asthma cohort: a clinical trial to assess asthma exacerbation phenotypes. Am J Respir Crit Care Med 2022;206:545–53. 10.1164/rccm.202201-0129OC 35549845 PMC9716911

[39] Diver S, Haldar K, McDowell PJ, et al. Relationship between inflammatory status and microbial composition in severe asthma and during exacerbation. Allergy 2022;77:3362–76. 10.1111/all.15425 Available: https://onlinelibrary.wiley.com/toc/13989995/77/11 35778780

[40] Diagnostics.S.Sight OLO. Sight OLO. 2023. Available: https://sightdx.com/en/product

[41] Heaney LG, Busby J, Bradding P, et al. Remotely monitored therapy and nitric oxide suppression identifies nonadherence in severe asthma. Am J Respir Crit Care Med 2019;199:454–64. 10.1164/rccm.201806-1182OC 30339770

[42] Kovesi T, Giles BL, Pasterkamp H. Long-term management of asthma in first nations and Inuit children: a knowledge translation tool based on Canadian paediatric asthma guidelines, intended for use by front-line health care professionals working in isolated communities. Paediatr Child Health 2012;17:e46–64. 23904776 PMC3448548

[43] Martin MJ, Beasley R, Harrison TW. Towards a personalised treatment approach for asthma attacks. Thorax 2020;75:1119–29. 10.1136/thoraxjnl-2020-214692 32839286

[44] Blakey J, Chung LP, McDonald VM, et al. Oral corticosteroids stewardship for asthma in adults and adolescents: a position paper from the thoracic society of Australia and New Zealand. Respirology 2021;26:1112–30. 10.1111/resp.14147 34587348 PMC9291960

[45] Dellit TH, Owens RC, McGowan JE, et al. Infectious diseases society of America and the society for healthcare epidemiology of America guidelines for developing an institutional program to enhance antimicrobial stewardship. Clin Infect Dis 2007;44:159–77. 10.1086/510393 17173212

[46] Juniper EF, O’Byrne PM, Guyatt GH, et al. Development and validation of a questionnaire to measure asthma control. Eur Respir J 1999;14:902–7. 10.1034/j.1399-3003.1999.14d29.x 10573240

[47] Juniper EF, Svensson K, Mörk A-C, et al. Measurement properties and interpretation of three shortened versions of the asthma control questionnaire. Respir Med 2005;99:553–8. 10.1016/j.rmed.2004.10.008 15823451

[48] Mahler DA, Wells CK. Evaluation of clinical methods for rating dyspnea. Chest 1988;93:580–6. 10.1378/chest.93.3.580 3342669

[49] Traister RS, Fajt ML, Landsittel D, et al. A novel scoring system to distinguish vocal cord dysfunction from asthma. J Allergy Clin Immunol Pract 2014;2:65–9. 10.1016/j.jaip.2013.09.002 24565771

[50] van Dixhoorn J, Duivenvoorden HJ. Efficacy of Nijmegen questionnaire in recognition of the hyperventilation syndrome. J Psychosom Res 1985;29:199–206. 10.1016/0022-3999(85)90042-x 4009520

[51] Durrington HJ, Gioan-Tavernier GO, Maidstone RJ, et al. Time of day affects eosinophil biomarkers in asthma: implications for diagnosis and treatment. Am J Respir Crit Care Med 2018;198:1578–81. 10.1164/rccm.201807-1289LE 30156881 PMC6298638

[52] Van Rossem I, Hanon S, Verbanck S, et al. Blood eosinophil counts in chronic obstructive pulmonary disease: adding within-day variability to the equation. Am J Respir Crit Care Med 2022;205:727–9. 10.1164/rccm.202105-1162LE 34797749

[53] Antosova M, Bencova A, Psenkova A, et al. Exhaled nitric oxide - circadian variations in healthy subjects. Eur J Med Res 2009;14:6–8. 10.1186/2047-783x-14-s4-6 20156715 PMC3521365

[54] Harris PA, Taylor R, Thielke R, et al. Research electronic data capture (Redcap)--a metadata-driven methodology and Workflow process for providing translational research informatics support. J Biomed Inform 2009;42:377–81. 10.1016/j.jbi.2008.08.010 18929686 PMC2700030

[55] Harris PA, Taylor R, Minor BL, et al. The Redcap consortium: building an international community of software platform partners. J Biomed Inform 2019;95:103208. 10.1016/j.jbi.2019.103208 31078660 PMC7254481

[56] Thwaites RS, Jarvis HC, Singh N, et al. Absorption of nasal and bronchial fluids: precision sampling of the human respiratory mucosa and laboratory processing of samples. J Vis Exp 2018:56413. 10.3791/56413 29443104 PMC5908664

[57] Bafadhel M, McCormick M, Saha S, et al. Profiling of sputum inflammatory mediators in asthma and chronic obstructive pulmonary disease. Respiration 2012;83:36–44. 10.1159/000330667 21912093 PMC3417284

[58] Bonini M, Di Paolo M, Bagnasco D, et al. Minimal clinically important difference for asthma endpoints: an expert consensus report. Eur Respir Rev 2020;29:190137. 10.1183/16000617.0137-2019 32499305 PMC9488652

[59] Statistics Canada. Focus on geography series, 2016 census - census metropolitan area of Sherbrooke. 2019. Available: https://www12.statcan.gc.ca/census-recensement/2016/as-sa/fogs-spg/Facts-cma-eng.cfm?LANG=Eng&GK=CMA&GC=433&TOPIC=7 [Accessed 13 Jan 2022].

[60] Benjamini Y, Hochberg Y. Controlling the false discovery rate: a practical and powerful approach to multiple testing. J R Stat Soc Ser B 1995;57:289–300. 10.1111/j.2517-6161.1995.tb02031.x Available: https://rss.onlinelibrary.wiley.com/toc/25176161/57/1

[61] Aaron SD, Vandemheen KL, FitzGerald JM, et al. Reevaluation of diagnosis in adults with physician-diagnosed asthma. JAMA 2017;317:269–79. 10.1001/jama.2016.19627 28114551

[62] Li G, Taljaard M, Van den Heuvel ER, et al. An introduction to multiplicity issues in clinical trials: the what, why, when and how. Int J Epidemiol 2017;46:746–55. 10.1093/ije/dyw320 28025257

[63] Howell I, Mahdi M, Bafadhel M, et al. Recovery of breakthrough asthma attacks treated with oral steroids while on monoclonal antibody therapy: protocol for a prospective observational study (BOOST). JMIR Res Protoc 2023;12:e46741. 10.2196/46741 37351918 PMC10337461

[64] Bafadhel M, McKenna S, Terry S, et al. Blood eosinophils to direct corticosteroid treatment of exacerbations of chronic obstructive pulmonary disease: a randomized placebo-controlled trial. Am J Respir Crit Care Med 2012;186:48–55. 10.1164/rccm.201108-1553OC 22447964 PMC3400995

[65] Ramakrishnan S, Jeffers H, Langford-Wiley B, et al. Point of care blood eosinophil guided oral prednisolone for COPD exacerbations: a multi-centre double blind randomised controlled trial (the Starr2 trial). Eur Respir J 2022;60. 10.1136/thorax-2022-BTSabstracts.5 37924830

[66] Ramakrishnan S. Prednisolone for COPD exacerbations: time for a rethink. ERJ Open Res 2023;9:00464-2023. 10.1183/23120541.00464-2023 37701365 PMC10493706

